# A survey of the training experiences and needs on Wellcome Trust PhD programmes

**DOI:** 10.12688/wellcomeopenres.19561.1

**Published:** 2023-10-09

**Authors:** Charlotte Fawcett, Kathryn Sandilands, Rispah Ng'ang'a, William Muasya, Ieva Budriunaite, Humma Andleeb, Winfred Gatua, Laetitia de Abreu Nunes, John Oketch, Giulia G. Piazza

**Affiliations:** 1 University of Leicester, Leicester, England, UK; 2 University of East Anglia, Norwich, England, UK; 3 Quadram Institute Bioscience, Norwich, England, UK; 4 University of Glasgow, Glasgow, Scotland, UK; 5 University of Dundee, Dundee, Scotland, UK; 6 University College London, London, England, UK; 7 University of Bristol, Bristol, England, UK; 8 Queen Mary University of London, London, England, UK

**Keywords:** PhD, training, questionnaire, research culture, doctoral training programme

## Abstract

**Background:**

Training for PhD researchers was previously identified by the Wellcome Trust funded Emerging Research Cultures project as an area for further investigation to ensure an inclusive culture which enables PhD students to become well-rounded researchers.

**Methods:**

The Taskforce on Training conducted a survey of 36 Wellcome Trust-funded PhD students and 10 programme administrators to evaluate the provision of training in eight key areas. This survey examined a number of issues, such as availability and knowledge of training, potential gaps in training, and the perceived usefulness of training.

**Results:**

PhD students reported that training was generally useful and viewed as important; technical training in particular was highly valued. However, the survey identified that students desired additional training in project management and personal development. A survey of programme administrators highlighted the wide variety in training availability for students across several Wellcome Trust programmes currently run in the UK.

**Conclusion:**

In response to these findings, a number of recommendations were made. These included: promotion of peer mentoring for PhD students, and alternative methods for delivery of well-being training. However, this report only explores the views of a limited number of Wellcome Trust funded PhD students and would benefit from further research into the experiences of PhD students, programme administrators, and PhD supervisors.

## Introduction

### Background

In 2020, the Emerging Research Cultures (ERC) project was launched to share the practices and experiences of research culture across Wellcome Trust funded PhD programmes that have started since 2019. Research culture generally includes ideas such as equality and diversity, mental health, bullying and harassment and other aspects such as encouraging good scientific values which can, in turn, help to build a successful and engaging research community. The project was launched to identify positive actions and challenges faced by researchers in differing work environments, to allow for the critical evaluation of current practices and the promotion of a healthy research culture.

Shortly after the project launch, group discussions were held with both Wellcome Trust funded PhD students and staff from 18 of the 23 UK-based Wellcome Trust funded PhD programmes at 15 institutions. The discussions considered several key concerns including; the ongoing negative impacts of the COVID-19 pandemic on funding and career prospects, good supervisory practices, the role of flexible working and mental health for researchers. Upon the conclusion of these group discussions in 2021, a report was published in 2022 ( Carusi *et al.*, 2022a) highlighting four primary areas of interest in which further work was needed.

One of the key areas identified by Carusi *et al.* (2022a) was training. Students felt that they lacked training in non-technical skills such as grant writing, whilst staff acknowledged a lack of training in people management skills and mental health awareness. Consequently, in June 2022 the Taskforce on Training (hereafter referred to as the taskforce) was formed consisting of first- and second-year PhD students across Wellcome Trust funded PhD programmes. The research undertaken by the taskforce is therefore reflective not only of the experiences of study participants but is also driven by the experiences of the PhD students who created the questionnaire and performed the subsequent analysis. The taskforce specifically focused on what training is delivered to students and in what format, and how training opportunities are promoted in their institutions. Moreover, it also assessed students’ level of satisfaction with training and where students felt improvements were needed. More information about the Emerging Research Cultures Project and its generated taskforces is available here: https://interchangeresearch-my.sharepoint.com/:p:/g/personal/erc_inter-changeresearch_com/EbKwLVIeOu5MswTKLI3tVnQBGTBBxRt5aJCGOOR_WAkHyQ?e=wtCV6S

The following report summarises the findings and recommendations of the training taskforce from the ERC project. It can be used to reflect on current training practices across programmes, their successes and concerns, and be used to standardise or implement training to ensure that every Wellcome funded PhD student has the skills required to become a well-rounded researcher. Although this report is specific to Wellcome-funded projects, the recommendations made may be of benefit to other PhD programmes.

### This research in context

These findings are consistent with recent scholarly work concerning the training needs of PhD students. A previous study of postgraduate researchers from a wide range of disciplines demonstrated that a majority of PhD students would like more training to be integrated into their programme of study ( Jones, 2017). This accords with an increasing appreciation of the fact that PhD students, upon graduation, will not necessarily enter academia: they will follow a wide range of career paths ( Gould, 2015; Jones, 2017 and Woolston, 2017) and so need diverse, transferable skills ( Doonan *et al.*, 2018; Jones, 2017). In particular, personal development training was specifically highlighted in previous work as an area in which students felt that they had not received sufficient support ( Jones, 2017).

Although the survey of Wellcome Trust PhD students drew from a relatively small cohort, it is clear that the training experiences and needs expressed by participants are reflective of a broader perception of training in research degree programmes. The Wellcome Trust studentships which inspired this research are therefore representative of the practices which constitute doctoral training programmes more widely.

### Aims and objectives

The taskforce began by evaluating the training needs of the PhD students. This process included identifying key areas of interest, such as gaining professional skills which play an important part of students’ developing academic portfolio. The taskforce further laid down a research framework on how to approach research on this topic with consideration of the expected research outputs, culminating in the production of this report.

The first concern of this taskforce was to identify students’ current thoughts about the training they had received, as it may differ between cohorts due to differing years of study and the effects of the COVID-19 pandemic as summarised by Carusi *et al.* (2022b). Upon completion of these early discussions, the initial aims for the taskforce were:

- To explore the training needs and experiences of staff and students across 23 Wellcome Trust funded programmes.
- To create a formal report describing the findings of those training needs and experiences which could be used as guidance to standardise training across all programmes.

To achieve these aims, surveys (see *Extended data*, ( Fawcett *et al.*, 2023b)) were conducted of students and administrators to identify the training currently available, training students found most useful, how training was delivered, and areas of training students felt were lacking. Students on Wellcome Trust doctoral training programmes across the UK were invited to take part in the study in August and September 2022, drawing from the following institutions: King’s College London, Queen Mary University of London, University College London, University of Bristol, University of Cambridge, University of Dundee, University of East Anglia, University of Edinburgh, University of Glasgow, University of Leicester, University of Nottingham, University of Oxford, and University of Sheffield. Invitations to participate were issued through emails from the ERC project which were circulated by programme co-ordinators. The survey was accessible by a link leading to a Microsoft Office form, which students could complete by both selecting responses on a sliding scale and by offering free text comments to some questions. 36 students participated in the survey, although not all questions were mandatory and some students elected not to respond to all questions.

### Early findings

The objectives and the initial intentions of the taskforce with regard to data collection were presented at the Wellcome Trust PhD Students’ meeting in London for first- and second-year students in mid-July 2022. These students were current first- and second-year PhD students from 23 Wellcome Trust funded PhD programmes across the UK. In addition to describing the work of the taskforce to date, the delivery of this presentation also provided a valuable opportunity to engage a wider audience of students in Wellcome Trust programmes. This allowed the taskforce to gain feedback in the form of a Mentimeter ‘word cloud’ and additionally to recruit further members interested in pursuing these objectives.

During the presentation, the taskforce requested instinctual responses to the question, ‘how would you describe the training that you have received so far as a postgraduate researcher?’ This was intentionally phrased in an abstract manner to attract a range of answers, thereby providing a gauge as to the attitudes of students in each year group to training. Using free access to the Mentimeter website, students were able to virtually submit short answers which were then compiled in a ‘cloud’ format and shown to the audience during the presentation. All answers were entirely anonymous and thus able to be retained for further reflection.

The number of students who responded to this exercise differed by year groups, with 60 first-year students recording their responses, whilst 43 second-year students recorded their experiences on the word cloud. The number of individuals at the second-year event was 75, including Wellcome staff, whilst there were 105 attendees at the first-year event including Wellcome staff. This shows a similar rate of non-response amongst both year groups, with a response rate of 57%. The reasons for non-response are unknown as information about non-respondents was not collected.

**Figure 1.**
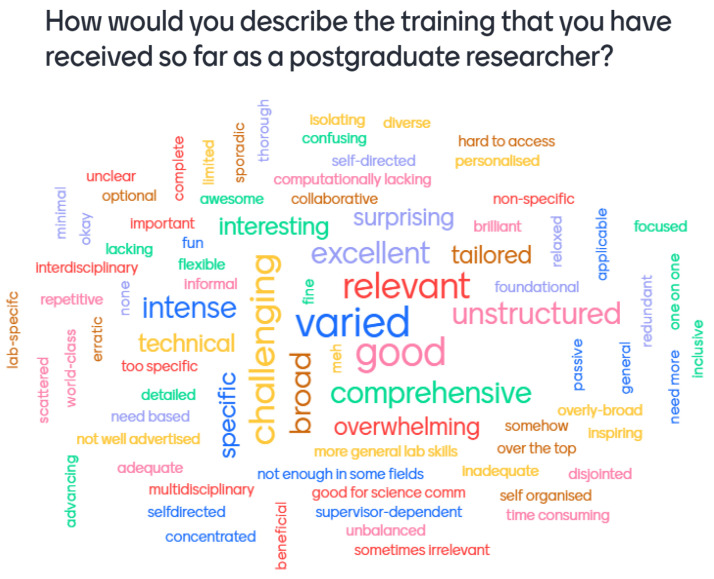
Mentimeter word cloud made up of responses from first-year students at the PhD Wellcome Meeting. Size of the word represents the number of times that idea was submitted by students during the online poll.

Responses, across both year groups, were varied and contained approximately equal numbers of phrases with connotations which were positive, negative, and neutral. Some examples of positive terms included: ‘brilliant’, ‘thorough’, ‘supportive’, or ‘helpful’. Other responses were negative, such as those which mentioned a sentiment that training was ‘not rigorous’, ‘unstructured’, or ‘sometimes irrelevant’. A great many responses also included neutral phrasing such as: ‘fine’, ‘interdisciplinary’, or ‘sufficient’. These responses indicated that whilst there are areas of training students felt happy about, there were areas of training which could be improved and these could be identified through a more comprehensive survey of student experiences regarding training. Concerns raised about the advertisement of training activities and access to information indicated an issue not indicated in the original discussion groups (see Figure 1). Nonetheless, this issue requires further investigation as it may suggest that students are not aware of training opportunities available to them at their institutions.

**Figure 2.**
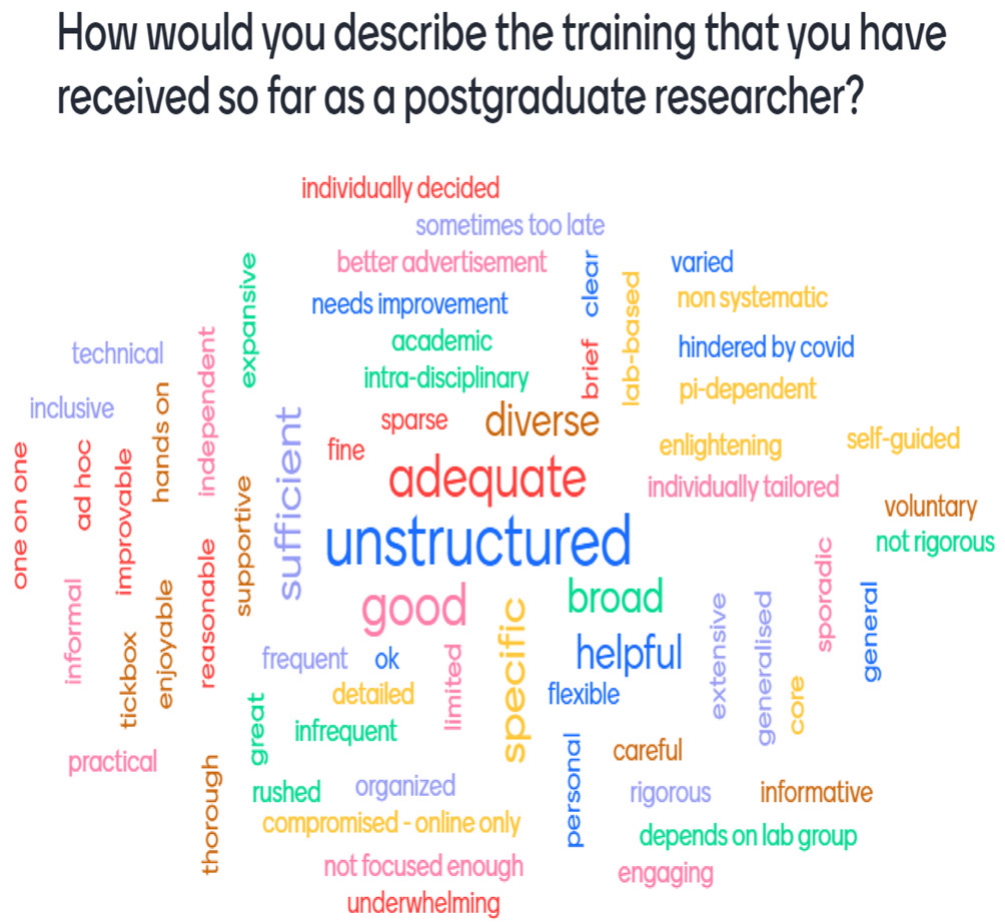
Mentimeter word cloud made up of responses from second-year students at the PhD Wellcome Meeting. Size of the word represents the number of times that idea was submitted by students during the online poll.

The software used for this poll, Mentimeter, changes the size of words in the word cloud to represent the number of times that word was submitted by the audience. Therefore, Figure 1 suggests that first-year students most commonly felt that their training was ‘varied’, ‘good’, ‘challenging’, ‘broad’ and ‘relevant’, all of which fall within the scope of positive terms. This suggests that first-year students present at the meeting had a positive experience of training. Figure 2, however, shows that second-year students most commonly felt that their training was ‘unstructured’, ‘adequate’, and ‘good’ with the terms falling across all three connotation groups; indicating that second-year students had more mixed training experiences. Consequently, it was important during our later surveys to record the year of study that the student was in, as this early investigation suggested that this would impact on the results obtained.

Immediately following the presentations at the event, the taskforce was able to engage in smaller group discussions with students interested in contributing to the proposed research. There was a high level of interest from attendants at the Wellcome events, with several students attending a drop-in session to find out more about the taskforce and its goal. This allowed for input from non-taskforce members about their concerns and queries around training and provided the taskforce with some new areas of interest when conducting their research. It also showed the importance that students felt this topic had.

Use of the word cloud format ensured a broad range of opinions could be reflected in the exploration of training experiences as the taskforce engaged in further research. It aided in the refining of questions for inclusion in the student experience survey later developed to investigate this topic in further depth. Furthermore, the Wellcome Trust events demonstrated the engagement of students with this topic and its importance to their future as researchers, in addition to highlighting differences between the training experiences of year groups, which will be of later interest to the taskforce.

## Methods

### Ethics

The research for this publication was conducted under the auspices of the Emerging Research Cultures Project Ethics Approval, and falls under its ethics protocol, as approved by an ad hoc ethics panel on 9th December 2020. The panel was comprised of three independent ethicists as the project did not fall under the remit of any institutional review boards. Written consent was obtained from participants prior to inclusion in the ERC community of practice, with an agreement that collected data could be used for future research up to 5 years following the conclusion of the Emerging Research Cultures project (currently funded until August 2025). The student survey was conducted after obtaining informed consent from participants and under ethics approval for the ERC project. However, as no personal data was collected from participants in the administrator survey, informed consent was not required. Prior to the release of the survey, all questions were approved by the ERC team.

### Surveys

Following the initial students' response on training experiences at the Wellcome Trust event in July 2022, the taskforce developed a novel student survey to further understand; 1) which areas of training students had received, 2) how training was delivered, recorded, and shared; and 3) areas in which students felt they may benefit from further training. Additionally, the authors of this paper conducted a novel survey on Wellcome Trust funded PhD programme administrators to establish the training opportunities currently available to students, which would help indicate students’ awareness of the training offered ( Fawcett *et al.*, 2023b). Moreover, it would also enable the taskforce to identify differences in training between the various programmes funded by the Wellcome Trust. The individuals surveyed were the main point of contact for each of the 23 Wellcome Trust funded PhD programmes; often the programme lead or a designated programme administrator. This survey was distributed by email and included a link to a Microsoft Excel spreadsheet for administrators to complete regarding different aspects of training available to their PhD students. Administrators were recruited by the ERC project, as part their community of practice ( Emerging Research Cultures, 2023). No personal information about administrators was collected; all answers pertained to the Wellcome Trust programme with which they were affiliated. No exclusion criteria was used.

For ease of recording, the administrator survey was divided into eight distinct areas of training. These eight areas were:

1) Professional development (including presenting or writing workshops);
2) Wellbeing and support (including mental health support and signposting);
3) Equality, diversity, and inclusion training;
4) Engagement and outreach (including science communication);
5) Personal development and management (including time management, research integrity, and expectations as a PGR);
6) Career development;
7) Cohort-specific training; and
8) Technical training (including training in a specific software or laboratory skills).

For each of these categories, administrators were asked to consider the source of the training provided; whether this represented a one-off session; how the training was delivered; the length of the training session; how the training was advertised; and when in the course of study this training was provided. Since the study was designed with the intention to compare the administrators’ survey findings with those of the student survey, the latter student survey was developed using the same eight training categories.

To adhere to General Data Protection Regulations (GDPR) ( European Parliament and Council of the European Union, 2016), the taskforce did not request that participants identify the Wellcome Trust programme on which they were enrolled during the surveys. However, it was necessary to ascertain the year group of students to analyse the appropriateness of the timeframe in which training occurred. Additionally, to comply with GDPR, information such as gender and age were not collected by the taskforce, which means that gender-specific analyses cannot be performed. The administrator survey also requested that the specific programme be identified for purposes of comparison, although this did not involve any personally identifiable information. For the survey questions, please see *Extended data* ( Fawcett *et al.*, 2023b). Where information about the programme name was gathered, this was pseudonymised. This data was processed and stored according to the ethical approval of the ERC Project, on secure servers subject to UK law.

The student survey investigated the availability, effectiveness and knowledge of training opportunities, amongst other points of interest. Students were asked to rate the usefulness of types of training and the appropriateness of the format, whether it be through a workshop, lecture, or online session. It was hoped that this would identify any training that was considered extraneous and also highlight potential means of improving engagement with training. For students, the survey was distributed via an email which included a link to a Microsoft form, where the questions were listed ( Fawcett *et al.*, 2023b). These students were also recruited as they were current students on one of 23 Wellcome Trust funded UK PhD programmes, with no exclusion criteria, at one of 15 UK-based institutions. Access was provided to these students through the Emerging Research Cultures community of practice ( Emerging Research Cultures, 2023).

Most questions were presented with answers available as either a five-point sliding scale or a simple “yes” and “no” format. This was intended to engage those surveyed by ensuring that the questionnaire could be completed relatively quickly and to allow for more efficient data analysis. Some questions, however, necessitated a more open-ended response. These included: “What training modules did you find most useful?”, “Are there any aspects of the PhD project or life as a PhD student which you do not feel equipped to manage?”, and “What training would you like to be offered?” As these questions specifically sought to understand the student experience and to engage with student opinions as to how training might be enhanced, it was necessary to allow participants the freedom to answer such questions fully.

Potential gaps in the training provision for PhD students were considered the most significant investigative aim of the survey, which necessitated hearing students’ own voices. This was possible through the Mentimeter word clouds ( Figure 1; Figure 2) and supplemented by the multiple-choice questions but required a limited number of free text answers to benefit from a wide range of opinions on training needs. The survey was divided into a series of sections relating to training: awareness, scheduling, content, format, usefulness, recording, and overall satisfaction. Both the student and administrator surveys were distributed via email from the ERC email account, alongside surveys from other ERC projects.

**Figure 3.**
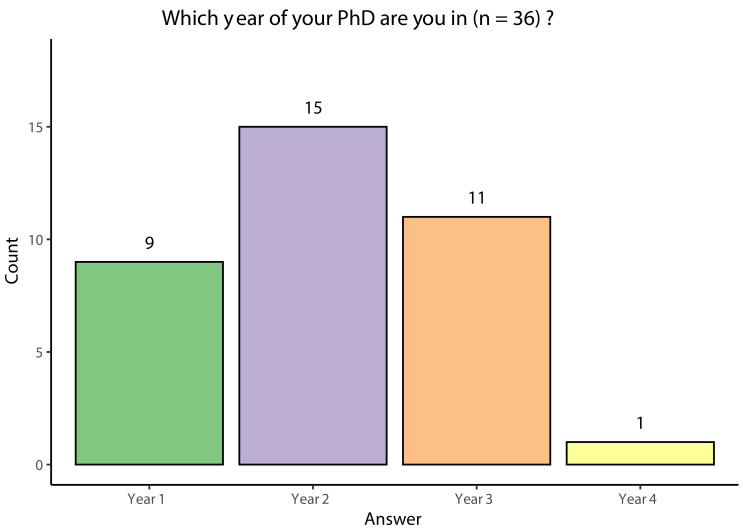
Bar chart showing the respondents' year of study.

Responses were obtained from 36 Wellcome Trust funded PhD students in different years of study at institutions across the UK ( Figure 3). As the institution of the participant was not recorded for GDPR reasons, it is unknown how many of the 23 UK based Wellcome Trust programmes were captured. Although the same survey was sent to all participants, some chose to answer the survey only partially. During analysis, missing data was not considered and instead each question was analysed separately, with all students who answered a specific question being included at each stage of analysis. Second year PhD students represented the largest number (15/36) of the respondents of the survey conducted. However, it is important to note that the survey was distributed in September and October 2022, during the start of the new academic year: as such, querying students about their year of study could yield different results depending on when precisely in that period they completed the survey. Additionally, there was a high level of non-response, even excluding the members of the taskforce, as a total of 180 first and second-year Wellcome Trust students had attended the Wellcome Trust events in July 2022. However, the reason for this cannot be determined as information about non-respondents was not collected.

### Analysis

After the completion of the survey, the data was anonymised and analysed. Analysis was conducted by the data analysis action group using R (version 4.1.0) and RStudio (Build 554). For the student survey, quantitative questions with multiple choice responses were analysed by tallying the number of responses for each outcome and plotting these as bar charts, dividing these results by year groups. Bar charts were generated using the R package ‘ggplot2’ (version 3.3.6). Bar charts were used because they can easily represent trends in the data such as the most popular option and are a visually appealing method of showing information.

Open questions were analysed by identifying and extracting the key themes from the answer, assigning this to a category and plotting these categories into bar charts. This allowed for answers to open questions to be explored via shared themes to identify key concerns and interests of the students.

The administrator survey did not include any quantitative questions and so the reporting on this survey was analysed using the same method as the student survey, classifying administrator responses by training category and plotting bar charts to show trends in different categories across programmes ( Fawcett *et al*., 2023a). This allowed for the taskforce to report areas of training delivered by the programmes, in addition to how and when this training is delivered.

## Results

### Student survey

The following section will discuss the results of the student survey (see *Underlying data*) by training section.

### Awareness of training

**Figure 4.**
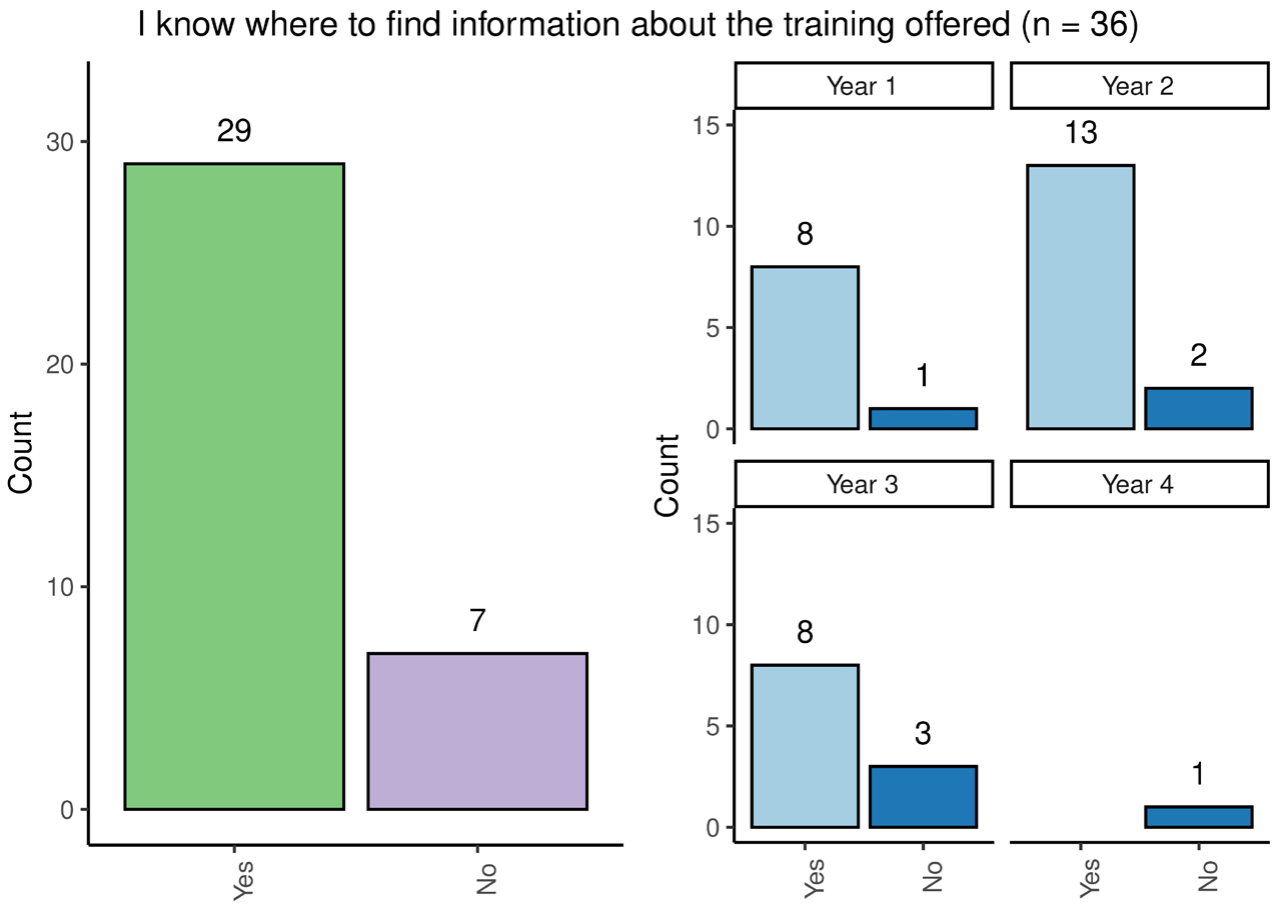
Bar chart showing overall responses regarding knowledge of where to find information about training offered. Reponses are broken down by year group.

One of the key areas of interest for the taskforce was student awareness of training opportunities. Although most participants agreed that they were informed about the upcoming training events, 19% did not know where to find the information on upcoming training opportunities ( Figure 4) ( Fawcett *et al.*, 2023a). Fewer participants in Year 3 of their studies knew where to find training than in other year groups.

When asked whether their Wellcome Trust PhD programme made them aware of training opportunities, only 52.7% (19/36) agreed with this statement. For first- and second-year PhD students, there was an approximately 50% split in responses, whilst third-year students reported that their programme had made them aware of available training opportunities 72.7% (8/11).

Overall, the data shows that most of the students participating in the survey were informed of the training opportunities available and knew where the find the relevant information.

### Scheduling of training

**Figure 5.**
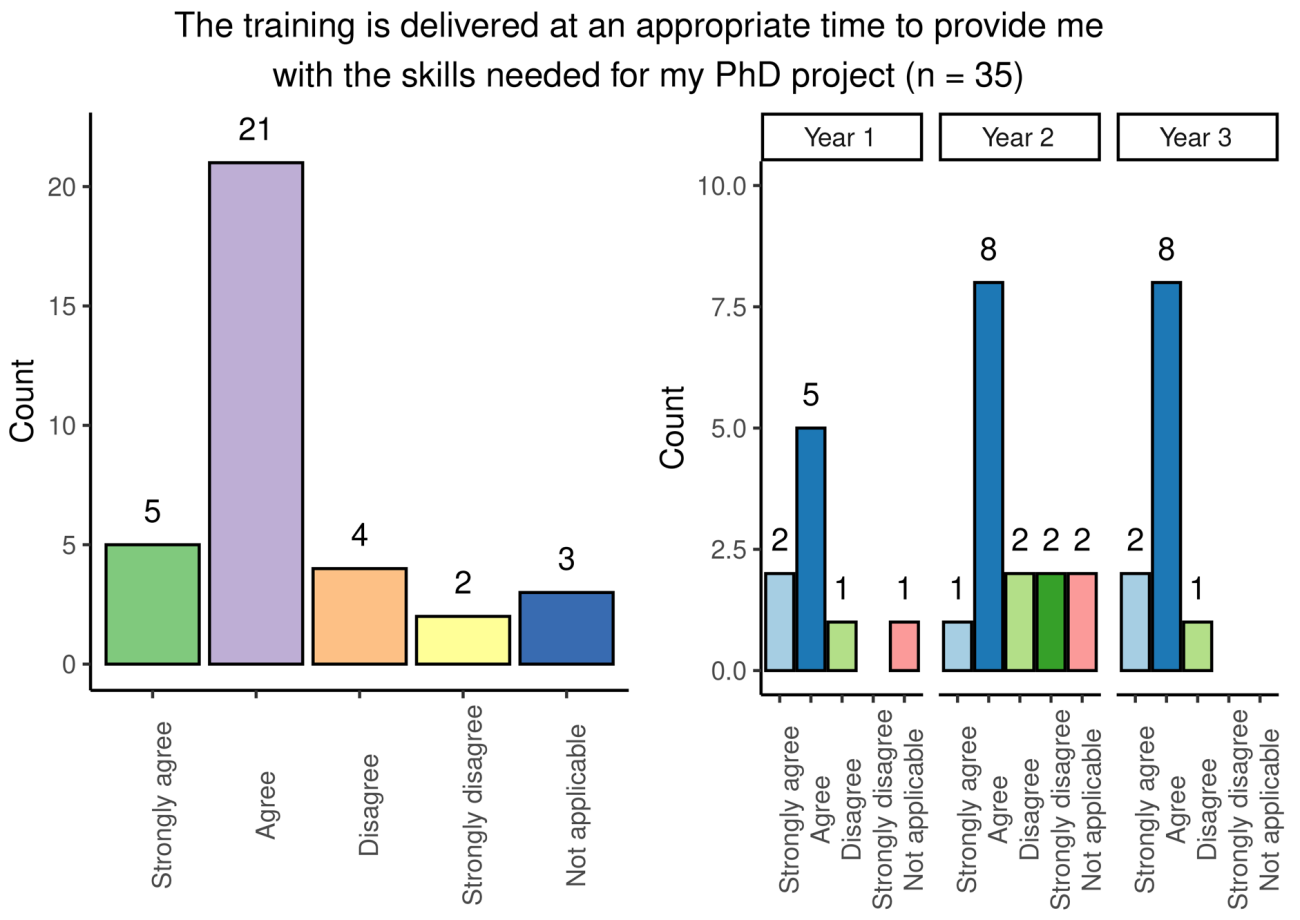
Bar chart showing responses regarding the appropriateness of timing of PhD training. Reponses are shown overall and broken down by year group.

A range of responses were obtained from students when questioned on whether their training was delivered at an appropriate time for use in the PhD project: responses were broadly positive and consistent across year groups, with the most popular response being ‘agree’ ( Figure 5). Second-year students had a marginally higher number of negative answers when compared with other year groups.

Although most students felt that the timing of training within the programme was appropriate, 57.6% (19/33) of survey participants stated that training was not repeated for students who have scheduling conflicts. This experience was generally consistent across the different year groups.

Overall, the findings show that most people agreed that training was provided at an appropriate stage in their studies but raised the need for regularly repeated sessions of training to account for scheduling conflicts.

### Content of training

Students were asked to reflect on the usefulness of training in the eight previously identified categories. Technical and cohort-specific training were both almost universally considered to have been useful across all year groups. More specifically, regarding technical training, the vast majority of students surveyed found the training to be useful with one single respondent finding it to be ‘extremely not useful’. Similarly, most students noted that cohort-specific training had been useful.

In contrast to an apparently high uptake of these forms of training, comparatively few students reported having received training on equality and diversity, reflected by a ‘not applicable’ response to the question of usefulness. Of 33 respondents, exactly half had not experienced this type of training. However, the students who had received equality and diversity training generally found it useful.

**Figure 6.**
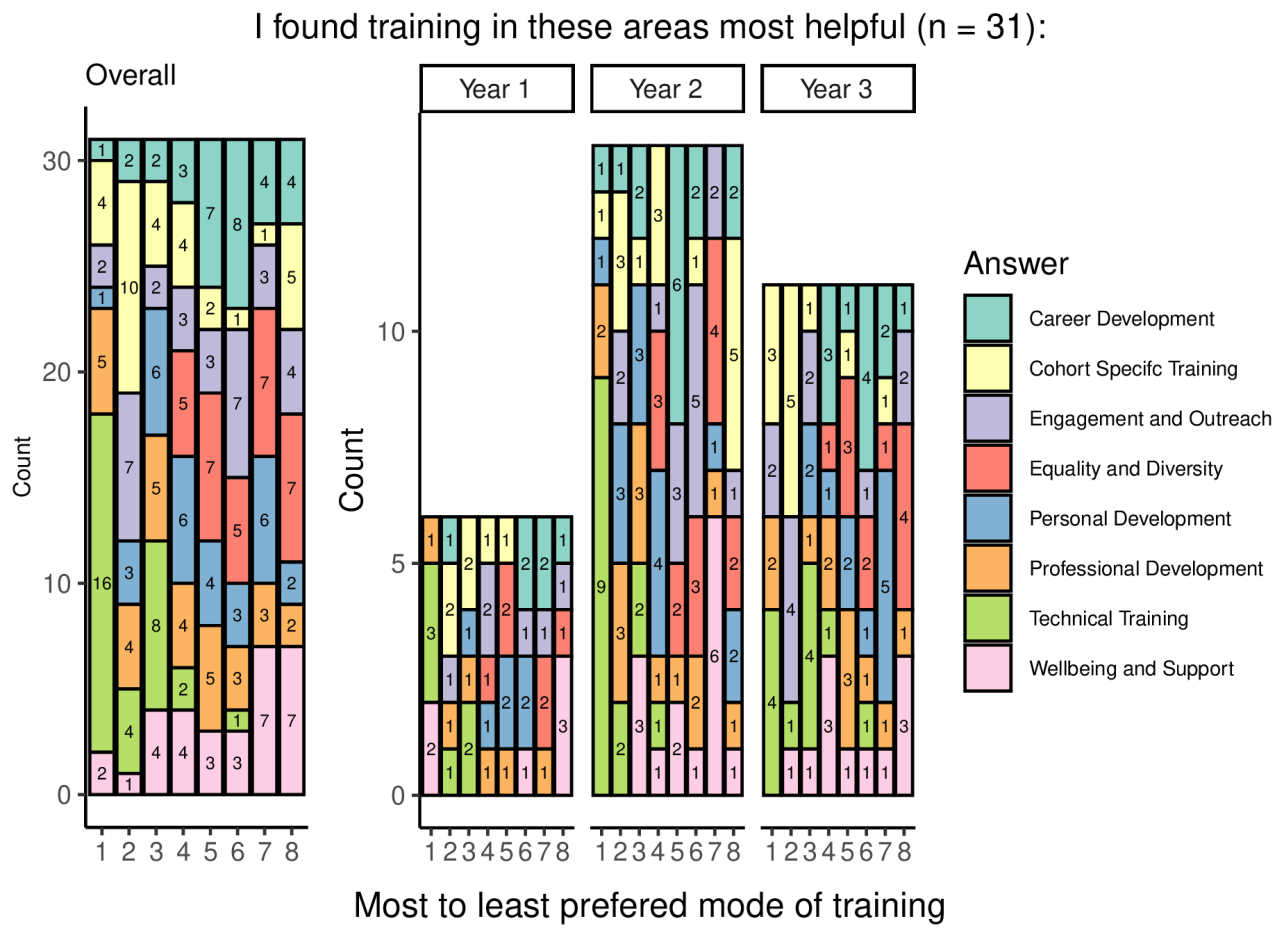
Graph showing the ranking of training categories from most to least useful. The graph shows the overall and year group breakdown ranking the eight categories of training from most to least useful, with the left-most columns being the most commonly chosen training categories. Numbers indicate the number of students who ranked the category for that level of helpfulness (e.g., 16 students ranked technical training ‘1’ or their ‘most helpful’ form of training).

In addition to being asked about the usefulness of the eight categories of training, students were also asked to rank them in order of helpfulness ( Figure 6). The overall results show that the most useful category of training was technical, with the second most commonly chosen category being professional development. The least useful training was commonly reported to be wellbeing training, and equality and diversity training. Third-year students displayed a more diverse range of the most popular training category than other year groups, but still indicated that technical training was the most useful.

Other forms of training saw similarly limited provision. Across the remaining five categories, a high proportion of respondents selected ‘not applicable’ answers regarding the helpfulness of this type of training: 28.1% (9/32) for engagement and outreach; 31.3% (10/32) for both professional development and personal development; 37.5% (12/32) for wellbeing; and as many as 53.3% (16/30) for career development. Where students had received training in these categories, their experiences were generally positive, with very few responses indicating that the training had not been helpful. In addition, 3 of 32 respondents found that personal development training had been ‘somewhat not useful’, 2 of 30 respondents stated the same for career development training, and one single response suggested that wellbeing training had been ‘somewhat not useful’. Overall, results suggest that some students may not have received training in these areas, but the majority of those who had, found that training was helpful.

**Figure 7.**
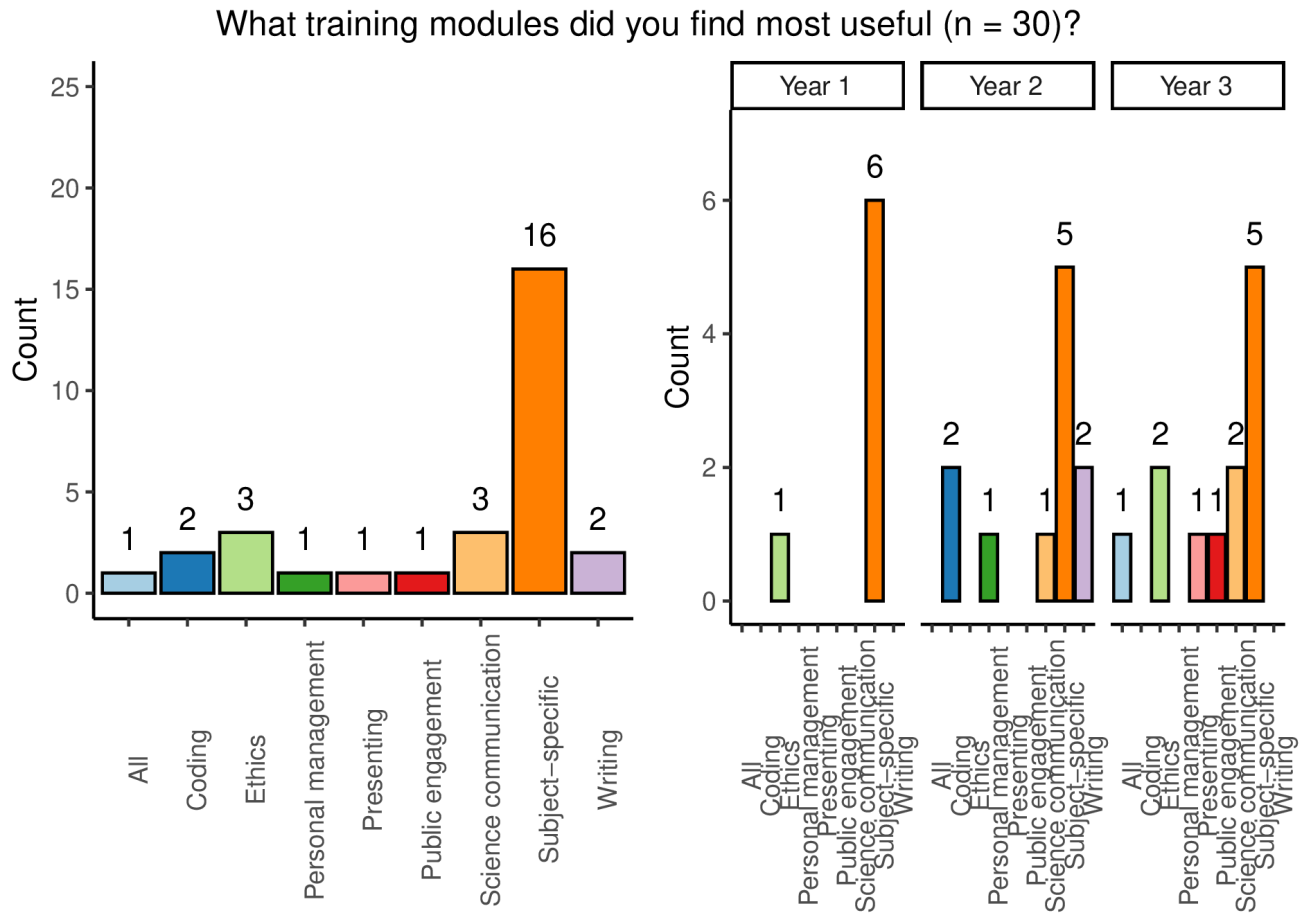
Bar chart showing responses to the question which training modules were most useful. Responses are shown overall and broken down by year group.

When asked to answer an open-ended question relating to the most useful training modules experienced across all year groups, subject specific training was overwhelmingly held to be the most useful, with 53.3% of respondents indicating that this was the case. Other modules mentioned included: coding, ethics, project management, presenting, public engagement, science communication, writing, or simply ‘all’ available training ( Figure 7). A majority of students (62.5%) reported that they had used skills learned in personal development training during the PhD, but a substantial remainder felt that they had not done so.

Students were given an opportunity to provide open answers to the question ‘are there any aspects of the PhD project or life as a PhD student you do not feel equipped to manage?’. 20 of the 33 respondents chose not to answer this question, while 3 stated that there were no concerns of this nature. Of the remaining 13 who answered ‘yes’, 11 gave detailed answers ( Fawcett *et al.*, 2023a). These responses raised a range of different issues. 3 students noted challenges relating to their wellbeing, which included struggles with ‘loneliness’ and ‘self-doubt’. There were 6 responses indicating issues related to academia or the PhD project, including concerns about selecting a supervisor, project management, and grant applications. A further 4 responses mentioned concerns regarding a career beyond research skills, raising issues such as struggles with networking, planning for next steps after the PhD, and managing finances.

Following on from the open-ended question regarding areas in which students ill-equipped to manage, respondents were given an opportunity to indicate types of training which they would like to be offered during their PhD. The following areas were identified: careers, ethics, financial training, grant or application writing, professional skills, project management, wellbeing, and subject specific training. While second-year students wished to see more subject-specific training, survey participants in the third year of their studies would like to see more training in project management.

Overall, the data shows that students were lacking in training on equality, diversity, and inclusion, although those who had received this training found it useful. Students also felt that their training in professional, personal, wellbeing, outreach & engagement, and career development was helpful, but some students reported that they had not had training in some of these areas. The most important training reported was technical and cohort- or subject-specific training that was consistent across year groups.

### Format of training

Training was reported to be generally compulsory, with 23 of 33 respondents stating that this is the case. Additionally, training was considered to be a good use of time and resources by 75.8% (25/33) of students participating in the survey; a further 15.2% responded that this was not applicable, and 9.1% disagreed with this sentiment.

**Figure 8.**
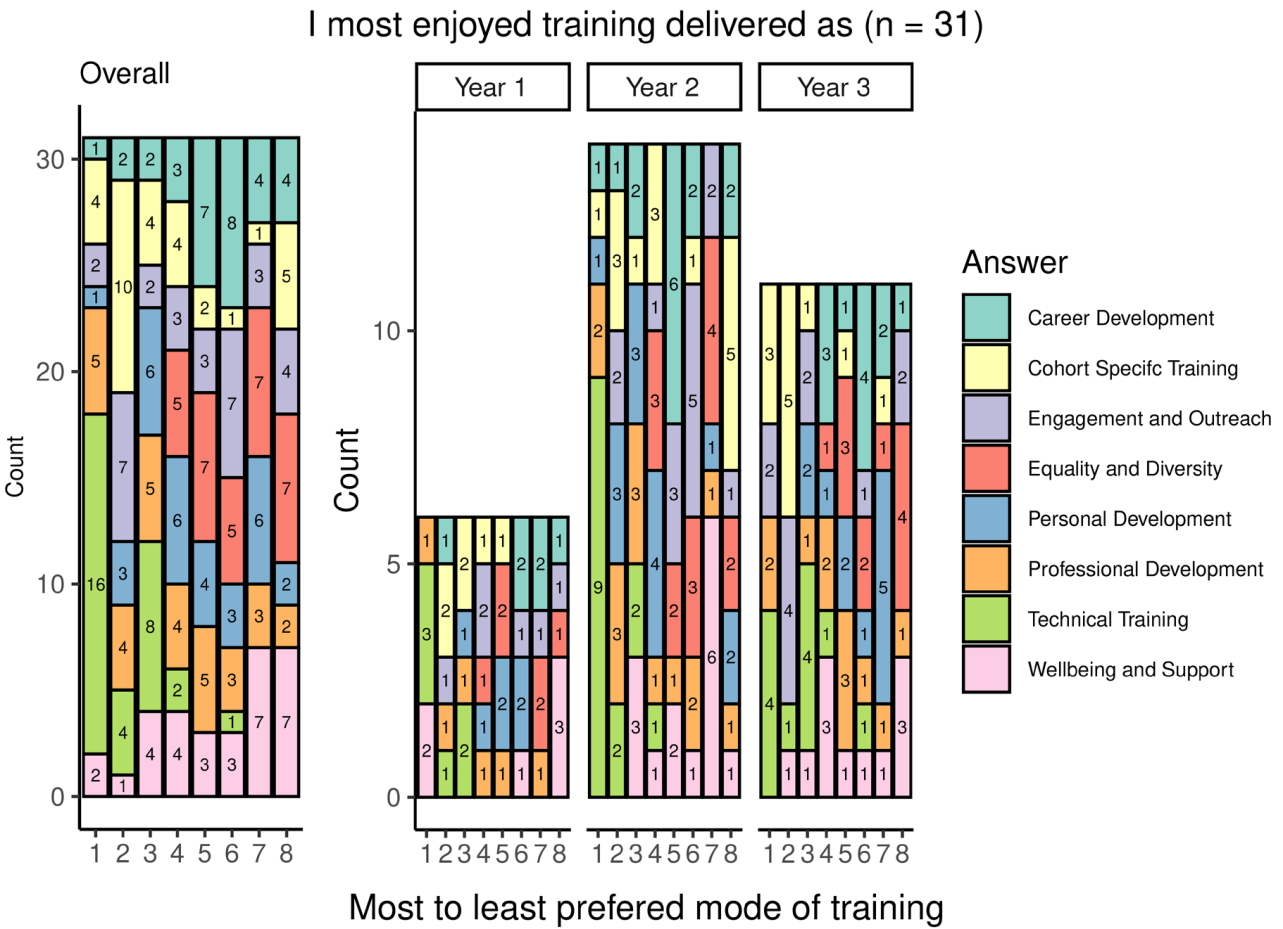
Graph showing the ranking of modes of delivering training from most to least enjoyed. The graph shows the overall and year group breakdown ranking seven modes of delivering training from most to least enjoyed. Numbers indicate the number of students who ranked the category for that level of enjoyment (e.g., 9 students ranked workshops ‘1’ or as their ‘most enjoyable’ form of training).

Students were asked to indicate their preferred mode of training and responses varied across the available options, which included: group discussions and activities; hands-on tutorials; lectures; mentoring; online training; required reading; and workshops ( Figure 8). The most commonly chosen types of favoured delivery method were workshops, hands-on tutorials and group discussions and activities, whilst the least popular modes of training are required reading and mentoring. Most students felt that training was offered in an appropriate format, with 22 of 33 respondents selecting the answer ‘agree’ in response to the statement ‘I feel the format of the training is generally appropriate to the subject matter’. Only two respondents disagreed with the statement.

Overall, the findings for this section were that most students felt that the delivery method used was appropriate for students and was mostly reported to be compulsory. Students also reported that training was considered a good use of time and resources.

### Overall satisfaction

Students were finally queried as to their overall satisfaction with their training during their PhD programme. Firstly, they were asked whether the training provided to them prepared them to become a good researcher, with 87.5% (28/32) respondents agreeing or strongly agreeing with this statement. This statement was only disagreed with by 4 students, all of whom were in second year. Students were then asked whether their PhD training had prepared them for life outside of academia, with 75% (24/32) of students agreeing or strongly agreeing to this statement. Again, students in the second year made up the majority of students disagreeing with this statement.

Students were asked as to their main objectives when engaging with training in an open-answered question. Most students reported ‘learning relevant skills’ to be their main objective, although other themes identified were: becoming a better researcher, open-mindedness, and personal development. Finally, students were given an opportunity to provide any final comments on training. Only five students responded, with three suggesting that their training had been good but incomplete, one reporting that they would prefer more in-person training, and a final student suggested that sharing training course information across Wellcome Trust-funded PhD programmes would be beneficial.

In summary, students reported that they were satisfied with their training and feel it will prepare them for life in research. Students also reported wanting to learn new and PhD-relevant skills when undergoing training. However, second-year PhD students reported that they did not believe that the skills they were learning would be transferable outside of academia.

### Administrator survey

As part of this project, a survey presented in a Microsoft Form was sent to PhD programme administrators across 23 Wellcome Trust-funded PhD programmes across 15 UK-based institutions. Ten responses were received and will be described in this section ( Fawcett *et al.*, 2023a).

**Figure 9.**
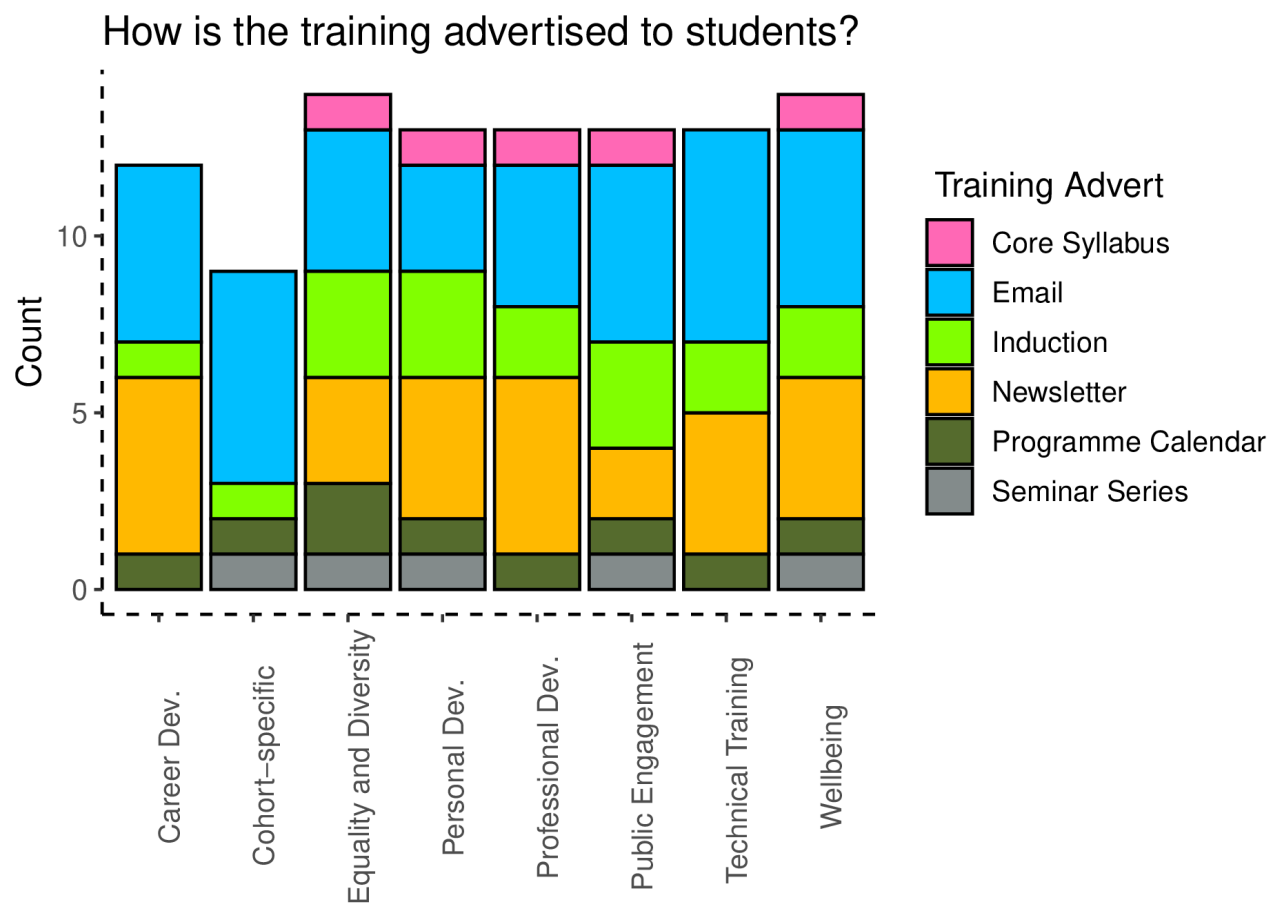
Bar chart showing the providers of the eight different categories of training.

Administrators were asked about training providers for the eight categories of training segregated in the student survey, with training being offered through the university, PhD programme, department or externally ( Figure 9). Equality, diversity and inclusion training was most commonly delivered through the university, whilst cohort-specific training was least commonly delivered through the university and was one of the training categories most commonly delivered through the PhD programme. Only three of the categories reported using external providers to supplement training, in professional development, technical training and wellbeing.

Administrators were also asked whether training was delivered through a one-off session, with responses across all eight categories being mostly evenly split between yes and no, with some programmes reporting that the question was not applicable or that it varied depending on the actual training session. For example, of nine administrators who answered this question regarding wellbeing training, one indicated that this was not applicable, one indicated that this varied depending on the session, two answered that the training was not repeated, and five stated that it was repeated.

The timing of training during the academic year and PhD student progression was also reported on. Although some types of training were reported to take place at the beginning of the academic year e.g., public engagement, equality, diversity and inclusion, technical and wellbeing training, training was generally reported to take place throughout the year and to vary. Administrators also reported that training for all categories except for careers development training was delivered in the first year of study, although training was generally reported to take place in all four years of study. Equality and diversity training had the most mixed reporting, with most programmes reporting it happened in first year, others reporting it happened after first year or in all years of study and two programmes reporting that the question was not applicable.

The mode of training delivery was also considered, including seminars, workshops and self-taught modules. Other forms of delivery were also mentioned, such as that careers development training could be delivered through industrial training partnerships. The results found that for all eight categories of training, the mode of delivery was diverse, with most training being delivered through varying methods.

Next, administrators were asked about the duration of training, which again varied between PhD programmes. Many of the training categories reported that the duration of training varied, although only equality and diversity training and wellbeing training was reported to be held in sessions lasting between 0-1 hour.

Lastly, administrators were queried about how training was advertised to students, which included being part of the programme syllabus, being included in emails, newsletters, or the programme calendar. Information about training across these categories was delivered using a range of methods, with dissemination through email being the most common response, followed by inclusion in a newsletter.

In summary, the output from the administrator survey was highly variable by programme, demonstrating a lack of consistency between programmes in delivering training to students. Equality, diversity, and inclusion training were often reported to vary between programmes, more so than other training types.

## Discussion

### Interpreting the survey findings

Firstly, it should be noted that whilst this study identified some areas in which training provisions may need to be reconsidered to meet PhD student needs, it was nearly universally agreed that training was a good use of time and resources. When reporting on training previously received, respondents appeared to recognise that provision had been generous and beneficial. This reflection emphasises the significance of the taskforce’s work to assess the training needs and experiences of students engaged in Wellcome Trust-funded PhD programmes. Training is evidently considered to be generally useful and important by the respondents to this study. Tailoring this training to account for student experiences to date and identified gaps in training needs could therefore aid in the further development of these and other students engaged in Wellcome Trust PhD programmes.

Students were asked to reflect on the usefulness of the training they had received, and technical training was particularly identified as being widely provided and was acknowledged to have been especially useful to students. Questions pertaining to the usefulness of training also highlighted perspectives on supporting wellbeing as responses were somewhat contradictory. A large percentage of respondents indicated that they had not received wellbeing training. Those who had reported that it had generally been useful. When presented with an opportunity to provide free text comments regarding aspects of the PhD project or life as a PhD student which they did not feel equipped to manage; three respondents indicated wellbeing concerns such as struggles with ‘loneliness’ and ‘self-doubt’. When asked what type of training they would like to be offered, two respondents suggested wellbeing training. Despite this, wellbeing training was not prioritised when students were asked to rank the helpfulness of various types of training: it was consistently rated as the least useful. This lack of prioritisation may suggest that while students seek some greater provision of wellbeing support, this should not necessarily take the form of training.

Of those who responded to the open-ended question regarding aspects of the PhD which they do not feel prepared to manage, a significant number of students indicated areas of personal development such as project or financial management or future planning. However, when asked whether they have used any personal development training to date, a third of respondents said that they had not done so. While this could appear to suggest that personal development training has not been useful, the lack of a ‘not applicable’ response for this question means that respondents may instead have been suggesting that they haven’t received any such training. Alternatively, if in the earlier years of their PhD, students may have not yet had an opportunity to use these skills but may find them helpful in the future.

When considering training formats, students largely preferred hands-on approaches such as tutorials, workshops, or group discussions. It was felt that training was generally appropriate to the subject matter, meaning that a range of approaches are likely utilised. However, training which involved required reading or mentorship ranked particularly low in preferences. This may be in-keeping with the desire for hands-on approaches to training, but it should also be noted that there was no option to exclude any options from the rankings. As such, if students had not experienced a particular form of training or had experienced it only rarely, then this may have appeared unpopular whereas in reality it may not have been commonly provided. An example of this is peer mentoring, which was commonly ranked near to the bottom, despite other forms of interactive learning being much more highly ranked. This may suggest that students instead have no or limited experience with this kind of training, resulting in it falling to the bottom of the ranking by default. Providing an option which allowed participants to make clear that they have not received a particular form of training may have clarified this.

Training scheduling was generally reported to not be repeated, although there was no provision for students to say whether they had access to recordings of missed training at a later date. Although methods such as recording training can allow students to still access the material, it can affect the level of engagement students have with the content and may also affect the behaviour of the recorded students. Furthermore, if training is not repeated to account for scheduling conflicts, this may adversely affect specific groups of students due to the timing of training (e.g., all day events). This includes PhD students who may be carers, parents, suffer from chronic illness and others working more flexibly. Care must therefore be taken when arranging training for consideration of these students and ensuring that they have an opportunity to attend live training sessions. However, no individual circumstances were assessed during the survey to establish whether there had been specific difficulties in accessing training. An important consideration here is the increased use of virtual training methods during the peak of the COVID-19 pandemic ( Bashir *et al.*, 2021) which may have impacted year groups differently. For example, students beginning their PhDs in 2020 are likely to have received the majority of their first-year training online, whilst cohorts which began later may have had more opportunities for in-person training.

Students also described their experiences of recording training, with most students stating that they had access to a university-maintained training record. However, there are year group differences in whether students feel they can access those logs, with first year students more commonly reporting that they can access their logs and lower rates for second- and third-year students. This may be due to programmes changing how this information is conveyed to new students or may be due to over-confidence on the part of students who have not yet actually attempted to access their training log.

When students were queried about their overall experiences of training, most identified that their main goal of undergoing training was to develop relevant skills, although other reasons were identified. This links to the preference for technical training, as these would be classed as relevant skills to the PhD project. Additionally, the survey found year group differences when asked whether this training prepared them for life inside or outside academia. Dissatisfaction with these statements was highest in second-year students, which may be due to considering these skills but not yet having received training to support them e.g., careers training, which was not reported in the administrator survey to take place specifically during first year. This may affect students’ perception of their readiness for the future beyond their PhD studies and how their training is relevant to this. It is possible that restrictions imposed due to the pandemic may have impacted the training experiences of these students, as discussed in Carusi *et al.* (2022a).

Students also reported that they were made aware of training, although there were year group differences in the number of students who reported that Wellcome had made them aware of training opportunities. It can be speculated that student awareness for training comes from their programme administrators and leads, leading to potential confusion in students as to the source of training information. The findings that students were aware of training is supported by the findings from the administrator survey, in which upcoming training is included in programme emails, newsletters or on the programme calendar, suggesting a variety of methods to make students aware of training opportunities.

The administrator survey identified that individual programmes have huge variety in what training is delivered, when it is delivered, how it is delivered and the duration of training, although training in all eight categories is broadly delivered across the responding programmes. Despite this, the greatest variation is in wellbeing training and equality and inclusion training, with some programmes reporting that training in these areas is not applicable or reports the shortest duration of training for these categories. This suggests that these two categories are less prioritised by programmes than other forms of training, despite indicators that students are wanting to engage with wellbeing training, reporting in the student survey that they would like more training aimed at wellbeing. The overall lack of consistency across programmes could indicate some inequities in the experiences of students enrolled in Wellcome Trust programmes but may equally suggest that training is adapted to the needs of the specific cohorts. This flexibility could be beneficial where it ensures that training is attuned to the student’s own project and development.

### Other considerations

The results from this survey show a range of experiences from Wellcome funded PhD students, identifying areas where students feel well supported but also areas in which they feel more training is required. However, it is important to note that only a total of 36 students responded to the survey, from a total of 254 students who were sent the survey and 10 programme administrators of the 23 programmes. This shows a significant level of non-response to the survey, which needs to be considered and addressed in future work. It may also be the case that the sample was biased as survey respondents may have had a pre-existing interest in PhD training. Moreover, the small sample size can make it difficult to draw robust conclusions by year group, although it is possible to report observed trends. Due to a tight timeline, the study was limited in addressing uptake and future work should consider allocating more resource and a longer timeline to explore this area.

In addition, the timing that the survey was given out further complicates this issue as it was administered across September and October, during a time when many PhD students are transitioning between year groups. This means that students on the same cohort, depending on when they completed the survey, may have reported to be in separate year groups. In future surveys it would be more useful to ask students to select the start year of their PhD, rather than their current year of study. Furthermore, as surveys can make it difficult to obtain more detailed information regarding the experiences of PhD students, future work into this area could also include the use of interviews with participants to obtain a more comprehensive understanding of the PhD training experience alongside these survey results.

Furthermore, although a survey was used, the taskforce recognises the limitations of such methods, particularly in determining detailed experiences of PhD students. Surveys may have high levels of non-response, which appeared to be the case in this report, with only 36 student responses. This was a novel questionnaire developed by the taskforce but was not validated prior to use. However, this study could be used as a pilot to validate and improve the use of this questionnaire for future research into training provisions.

Although surveys of Wellcome Trust students and programme administrators have been recorded, staff who are supervising these students were not surveyed. To further tie together information about the training received by Wellcome funded individuals, a survey of supervisors should be conducted to determine what training supervisors have received to support students with their areas of concern and any support that supervisors themselves might require.

Consequently, future work into this area may include validation of this questionnaire, surveying of supervisors to obtain their views and training, use of interviews to obtain more detailed information, and a need to promote response rates from PhD students surveyed.

### Recommendations

Despite the limits of our report, the taskforce has identified the following recommendations for the Wellcome Trust regarding the training of PhD students:

- Continue to provide valued training in technical skills
- Consider alternative methods to provide wellbeing support, for example access to a counsellor in the place of wellbeing training
- Introduce additional training in project management and personal development
- Consider the impact of training through peer mentoring between students
- Hold focus groups with students to identify failures in wellbeing support and equality and diversity training and how this can be tackled by Wellcome
- Consider wider review of the training needs of both students and staff of different institutions
- Consider standardising common areas of training between different programmes

### Summary

In conclusion, surveying Wellcome Trust funded PhD students found that the majority of students were engaged with their training and found it useful and a way to develop important skills. However, some weaknesses were identified in wellbeing support and equality and diversity training which may need additional consideration. This report demonstrates the necessity of further research into the training experiences of PhD students, which would enable greater reflection on their needs and development.

## Data Availability

Figshare: ERC Taskforce 3 Underlying Data https://doi.org/10.6084/m9.figshare.c.6765435.v2 ( Fawcett *et al.*, 2023a) This project contains the following underlying data: •     Data file Taskforce on Training - Student Questionnaire Analysis (R analysis of student survey output) •     Data file Taskforce on Training - Administrator Questionnaire Analysis (R analysis of administrator survey output) •     Data file Taskforce on Training - Raw Data (Excel file containing the raw data from the student survey used for later analysis) •     Data file Taskforce on Training – Raw Data from Administrators (Excel file containing the raw data from the administrator survey used for later analysis) Figshare: ERC Taskforce 3 Extended Data https://doi.org/10.6084/m9.figshare.c.6765441.v3 ( Fawcett *et al.*, 2023b) This project contains the following extended data: •     Data file Taskforce on Training - Figures, Graphs for publication (Figures included in the final report) •     Data file Taskforce on Training - Administrator Questionnaire (Novel questionnaire developed for administrators) •     Data file Taskforce on Training – Student Questionnaire (Novel questionnaire developed for students) Data are available under the terms of the Creative Commons Attribution 4.0 International license (CC-BY 4.0).

## Software availability

Archived source code at time of publication: https://zenodo.org/record/8193126.
