# Supplementary material for: A survey of the training experiences and needs on Wellcome Trust PhD programmes
Source: Wellcome Open Res. 2024 Sep 3;8:432. Originally published 2023 Oct 9. [Version 4] doi: 10.12688/wellcomeopenres.19561.4 (PMC11252645; doi:10.12688/wellcomeopenres.19561.4)
Supplement: Peer review history

# Reviewer report: Reviewer response for version 3

**DOI:** 10.21956/wellcomeopenres.24818.r87382

**Published:** 2024-07-31

**Authors:** Emma Flores

**Affiliations:** 1 University of Michigan, Ann Arbor, Michigan, USA

No further comments to make.

Is the work clearly and accurately presented and does it cite the current literature?

Partly

If applicable, is the statistical analysis and its interpretation appropriate?

Not applicable

Are all the source data underlying the results available to ensure full reproducibility?

Yes

Is the study design appropriate and is the work technically sound?

Yes

Are the conclusions drawn adequately supported by the results?

Yes

Are sufficient details of methods and analysis provided to allow replication by others?

Yes

Reviewer Expertise:

Graduate education: professional development, diversity and inclusion, peer mentoring, and graduate student engagement. Program evaluation and assessment.

I confirm that I have read this submission and believe that I have an appropriate level of expertise to confirm that it is of an acceptable scientific standard.

# Reviewer report: Reviewer response for version 3

**DOI:** 10.21956/wellcomeopenres.24818.r87383

**Published:** 2024-07-16

**Authors:** Filomena Parada

**Affiliations:** 1 CeiED, Interdisciplinary Research Centre for Education and Development, Lusófona University, Lisbon, Portugal

Overall the changes the authors did to the manuscript, much improved it and, for the most part, adequately addressed the concerns I expressed in the previous round of review.

 There are a few minor issues the authors still need to address. I detail these issues below.

1. I suggest the authors revise the manuscript and check for typos and other minor edits. They must ensure there are no missing or repeated words.
2. **Methods**. The author(s) do not report the total number of staff administrators participating in the preliminary study. They state reaching out to the main point of contact for the 23 Wellcome Trust PhD-funded programmes. These programmes are hosted in 15 UK-based institutions. They also state both ERC events were attended by staff members who participated in the study, but they did not disclose how many. Furthermore, there is no information on how many staff administrators were contacted and asked to participate and/or collaborate in the study (either in its preliminary stage or the survey).
3. **Results**. I suggest the authors use horizontal bar charts for some of the figures and see if this option helps increase their readability. It is sometimes difficult to be sure to which bar the legend concerns. In some other graphs, the series numbers do not show.
4. **Results**. When reporting the results from the Administrators’ survey, the authors often use the expression “the programmes reported/reporting”. Programme administrators and not the programmes answered the questions in the survey.
5. **Discussion**. The authors tend to focus on the specific topic they are discussing (e.g. content of the training, scheduling of the training). The discussion would gain depth if the authors cross-referred the findings from the different types of questions from the administrators’ and the doctoral researchers’ surveys. Illustrative are the apparent contradictions in the doctoral researchers’ answers to the training on career and personal development, wellbeing and equality, diversity and inclusion. When reflecting on these answers (as the authors did) several explanations can be found. A better understanding of their meaning can be achieved when adding the authors' discussion about the administrators' answers. In combination, this shows different priorities, understandings, needs and concerns on the part of the doctoral researchers and the administrators. It also allows us to raise the question – as the authors did – if training is always the best option to address the specific needs of doctoral researchers, namely regarding wellbeing or career development.
6. **Discussion**. Based on the results reported, doctoral researchers attending different years of the PhD programme expressed also different views on the usefulness of the training they received to date. This is an interesting finding that could merit some additional considerations on the part of the authors. I understand the need to be cautious based on what they discuss in the section on Other Considerations (Limitations?). However, I would have liked to have read more on their take on the finding.

Is the work clearly and accurately presented and does it cite the current literature?

No

If applicable, is the statistical analysis and its interpretation appropriate?

Partly

Are all the source data underlying the results available to ensure full reproducibility?

Yes

Is the study design appropriate and is the work technically sound?

Partly

Are the conclusions drawn adequately supported by the results?

Partly

Are sufficient details of methods and analysis provided to allow replication by others?

Partly

Reviewer Expertise:

Research careers, early career researchers advocacy and policy, career guidance and development

I confirm that I have read this submission and believe that I have an appropriate level of expertise to confirm that it is of an acceptable scientific standard.

# Reviewer report: Reviewer response for version 2

**DOI:** 10.21956/wellcomeopenres.22902.r76862

**Published:** 2024-04-08

**Authors:** Filomena Parada

**Affiliations:** 1 CeiED, Interdisciplinary Research Centre for Education and Development, Lusófona University, Lisbon, Portugal

The topic of the study is interesting and relevant. Understanding the doctoral researchers' perceptions of their training and identifying gaps is an important topic beyond the Wellcome Trust PhD programmes. Additional relevance comes from the authors’ intention to compare the results from the doctoral researcher with the results from the programme administrators. However, in its current format, the manuscripts raised several concerns, some of which were highlighted in the previous rounds of review.

**1. Introduction**

 1.1. The present study has at its background the notion of research culture(s). In the introductory paragraph of the manuscript, the authors provide an overview of the concept that does not adequately reflect its depth and complexity. Over the years, several organizations or movements, such as the San Francisco Declaration on Research Assessment (DORA), the Wellcome Trust or the Coallition for Advancing Research Assessment (CoARA), have addressed the topic in statement positions, reports, or other policy-oriented documents. Some of the leading members of these organizations or movements also published journal articles focusing on the topic of the contemporary (hypercompetitive) research culture and the need for change. I suggest the authors look into these publications as they will help them better frame their study.

 1.2. It struck me as odd that at no point in the introductory section of the manuscript, the authors attempted to discuss the notion of doctoral training: What are its scope and aims? How has it changed over time? What are the main challenges doctoral training currently faces? For some time now, these questions have been framing discussions around doctoral training and important documents such as the Salzburg Principles, which have been updated several times since its publication in 2005, have provided insights into discussions and directions to consider when reflecting on the issue.

 1.3. The section on Aims and objectives includes information that is not strictly about the study aims or objectives. The authors included information that is about the procedure and that should be in the section on the Method. Also, how the section is structured obfuscates the aims of the study, which should be the first thing to be presented to the reader. These aims should be clear and provide a background to all the steps the authors took when designing and implementing the study.

 1.4. I suggest the authors rethink the structure of the manuscript. This will help remove some redundancy from the text. It will also help clarify the study aims and objectives as well as the study design and procedure. Specifically, what the authors have called “Early findings” appears to be an exploratory or pilot study whose findings were used to support the design of a following study. My suggestion is that the structure of the article (i.e., how the different sections are presented and the information included in each section) better reflects this two-step approach to the study.

**2. Method**

 2.1. The authors opted to use a narrative approach to their description of the study method that does not consider the typical subsections in a section on the Method (e.g., participants, instrument(s), data collection or analysis procedures). I suggest the authors reconsider the current structure of the section on the Method and better organize the information to increase clarity on what they have done, how and why. Important information about the different components of the method, for example, who the study participants were, is scattered across the sections, which makes the reporting hard to follow. What data the authors collect and how? How did the procedure for collecting and analysing the data address the study aim(s) and/or research questions? What instruments did they use to collect the data and which target population?

 2.2. The procedure the authors used to collect the data from the doctoral researchers differed from the procedure they used to collect the data from the Ph.D. programme administrators not just in terms of the type of data collected but on the instruments the authors used – at least, this is what I understood from their reporting. Regarding the type of data collected, the authors claim GDPR compliance restricted their ability to collect certain information from doctoral researchers. Wouldn’t anonymization of the data (which they did with the data they collected from the administrators) address the issue? No clear explanations are provided about why different instruments were used to collect data from the administrators and the doctoral researchers. Considering one of the aims was to compare doctoral researchers’ and programme administrators’ results, it makes no sense to me why paired data and other sociodemographic data (e.g., gender, Ph.D. programme attended, discipline or field of research) were not collected from both groups of participants. The information the authors chose to not collect from the surveyed doctoral researchers would also have been important to understand partial responses and missing data patterns -as the authors themselves admit. This is an important limitation of the study that requires additional consideration and reflection on the part of the authors. Furthermore, their decisions must be better explained, including the decision to send the survey at the start of the academic year and to include first-years doctoral researchers.

 2.3. Was 254 the total number of doctoral researchers attending Wellcome Trust PhD programmes that have started since 2019? If not, what was the total number of doctoral researchers attending a Wellcome Trust Ph.D. programme and what criteria presided to the selection of the 254 participants. Regardless, the response rate is quite low overall. Although acknowledging this as a limitation in the section on the Other considerations, no reflection on the reasons for such a low response rate is presented.

**3. Results**

 3.1. Overall, the section on the Results is clear and well-structured. The authors reported differently the results from the doctoral researchers’ and the administrators' surveys. Perhaps this has to do with the fact that there were fewer answers, and these answers were scattered, as the authors highlight. However, no section comparing the doctoral researchers with the administrators’ answers is included. The authors stated this as one of the study's aims. What were the main similarities and differences observed in the answers the doctoral researchers and the administrators gave to the survey? To what extent are these answers/the results comparable?

 3.2. Other minor issues:

 - When reporting doctoral researchers’ perceptions on the usefulness of the training they received, the authors discuss their results by relating them to the results of another study. I suggest all discussions are left to the section on the Discussion and in the Results section only the study results are reported.

 - Sometimes it is hard to read to which category each bar in the graph corresponds. Perhaps using a smaller size in the font would help increase readability.

**4. Discussion and Recommendations**

 4.1. When reflecting on the results, the authors make almost no references to the literature on the topic. It is unclear the extent to which the study results overlap, complement, or contradict previous research on the topic. There are several studies examining the training needs and priorities of doctoral researchers, including reports produced by the Wellcome Trust, that would have helped give additional depth to the results of the present study. Also, recently, Nature published several interesting articles addressing the wellbeing and work-life balance of early career researchers. I suggest the authors consider using these and other relevant publications to frame the discussion of their results. Similar considerations can be made to almost all topics in the discussion of the results (e.g., mentoring/peer mentoring, the impact of COVID-19 on training, professional and/or career development).

 4.2. Although mentioning career development as a category of training, no additional information is provided about its content or its specific results. No consideration of the motives making doctoral researchers choose to train at the Ph.D. level is included. Universities or research institutes' ability to attract and retain talent, including at the level of Ph.D. training, has recently started to permeate discussions around the need for reforms, including the reform of doctoral training and its assessment. It is unclear the extent to which the present study considered these discussions in its framing or used them to reflect and interpret findings.

 4.3. No reflection on the similarities/differences between the results from the survey answered by the doctoral researchers and the survey answered by the administrator is made. Also, no real conclusion summarizing the experience and main gaps in the training of doctoral researchers is provided.

 4.4. Over the years, several recommendations have been put forward that address many if not all of the topics focused by the present study. It is unclear why the authors have not considered them when discussing their results and proposing recommendations for improving doctoral training.

**5. Other important issues**

 5.1. The authors systematically refer to doctoral researchers as students. Granted, in the UK, doctoral researchers have the status of students. However, internationally, the acknowledgement of doctoral candidates as researchers at the initial stages of their training is growing. Any reflection on doctoral training, its purpose, scope, strengths, and limitations, should consider such good practice and acknowledge those researchers working at the doctoral level as researchers and not as students. This is a minor but significant issue as it goes directly into our understanding of what doctoral training is and what it means to acquire a Ph.D.

 5.2. When presenting the background of the study, the authors state that the Taskforce on Training consisted of first- and second-year doctoral researchers across Wellcome Trust-funded PhD programmes. Why only first- and second-year doctoral researchers were included in the task force and why were the early findings only shared with these researchers? Considering the aim of the study was to examine the experiences of doctoral researchers attending Wellcome Trust Ph.D. programmes, wouldn’t it make sense to also include in the task force and in the discussion of the early findings doctoral researchers that were more advanced in their training?

**6. Minor issue**

 6.1. When addressing the context of the research, the authors use the expression PGR without first defining it.

Is the work clearly and accurately presented and does it cite the current literature?

No

If applicable, is the statistical analysis and its interpretation appropriate?

Partly

Are all the source data underlying the results available to ensure full reproducibility?

Yes

Is the study design appropriate and is the work technically sound?

Partly

Are the conclusions drawn adequately supported by the results?

Partly

Are sufficient details of methods and analysis provided to allow replication by others?

Partly

Reviewer Expertise:

Research careers, early career researchers advocacy and policy, career guidance and development

I confirm that I have read this submission and believe that I have an appropriate level of expertise to confirm that it is of an acceptable scientific standard, however I have significant reservations, as outlined above.

## References

[1] : A survey of the training experiences and needs on Wellcome Trust PhD programmes. *Wellcome Open Research* .2023;8: 10.12688/wellcomeopenres.19561.2 10.12688/wellcomeopenres.19561.2 PMC1125264539021513

# Reviewer report: Reviewer response for version 2

**DOI:** 10.21956/wellcomeopenres.22902.r72700

**Published:** 2024-02-16

**Authors:** Emma Flores

**Affiliations:** 1 University of Michigan, Ann Arbor, Michigan, USA

Thank you so much for the opportunity to review the manuscript “ *A survey of the training experiences and needs on Wellcome Trust PhD programmes” *and to be able to provide the following comments.

- Understanding the training and professional development needs of PhD students continues to be an important area of research. I think this manuscript could benefit by providing a high-level summary of research from Australia and the United States that has addressed this same issue.
- There was no mention of this, but I wondered if there was any incentive provided to the survey respondents.
- I would love to see a table that summarizes the response rates in addition to being embedded in the text. This will make it easier for the reader to get a quick snapshot of the total population of students and administrators surveyed.
- Please clarify in the text how you are defining ‘mentoring’. When I think of mentoring, my first thought is faculty or advisor mentoring and in this manuscript, it seems that the focus is on peer mentoring. I would invite the authors to define this more precisely.
- I do not see the value and believe you should exclude the one student who responded from year 4. They are only represented in Figure 1, and I could not find further mention of findings from students in their fourth year.  
- I really struggled with Figure 6 on page 9 and Figure 8 on page 11. I think a separate stacked bar chart for each of the groupings would make this much easier to interpret.
- This work is in part motivated by the desire to understand the ongoing negative impacts of the COVID-19 pandemic, yet there was no real discussion about it except in relation to virtual training. I agree that future research should ask students to identify the year that they started their Ph.D. but I also wonder if it would be possible to define the years of each group (Years 1-4). This might indicate if these students were pre- or post-pandemic or both.
- One thing that I continued to question while reading the results and discussion was the inclusion of Year 1 students in this research. To what extent are students being oriented and exposed to training opportunities prior to the start of their first year? Would Year 1 students be able to see the full range of training that will be available to them during their programme? I ask because there is an expectation being made in this study that these Year 1 PhD students are going to be able to respond to a survey about their needs and training experiences. It is indicated on page 7 that this survey was distributed in September and October 2022, so I, as a reader, have to assume that Year 1 students did in fact start this same year. While these new students would be able to speak to their needs, I am not sure how much information they have regarding the opportunities that will be made available to them.  
- I would agree with the first reviewer to be cautious about your findings regarding EDI training. It seems that the survey did not allow students to rank the training that they had engaged in. As a result, the findings still seem to suggest to me, even after the first revision, that students do not value EDI training. I do not feel that you have the evidence to make that claim and would recommend stating that additional research is needed.

I hope that these comments will be useful and invite any questions if any of the above is unclear.

Is the work clearly and accurately presented and does it cite the current literature?

Partly

If applicable, is the statistical analysis and its interpretation appropriate?

Not applicable

Are all the source data underlying the results available to ensure full reproducibility?

Yes

Is the study design appropriate and is the work technically sound?

Yes

Are the conclusions drawn adequately supported by the results?

Yes

Are sufficient details of methods and analysis provided to allow replication by others?

Yes

Reviewer Expertise:

Graduate education: professional development, diversity and inclusion, peer mentoring, and graduate student engagement. Program evaluation and assessment.

I confirm that I have read this submission and believe that I have an appropriate level of expertise to confirm that it is of an acceptable scientific standard, however I have significant reservations, as outlined above.

# Reviewer report: Reviewer response for version 1

**DOI:** 10.21956/wellcomeopenres.21668.r68645

**Published:** 2023-11-08

**Authors:** Noémie Aubert Bonn

**Affiliations:** 1 Department of Healthcare and Ethics, Faculty of Medicine and Life Science, Hasselt University, Hasselt, Flanders, Belgium

Thank you for inviting me to review the manuscript “ *A survey of the training experiences and needs on Wellcome Trust PhD programmes*” submitted at Wellcome Open Research. The manuscript describes the results of a survey with Wellcome Trust-funded PhD students and programme administrators on their impressions of the provision of training in the Wellcome Trust programme and on several elements of the training itself.

 Overall, the manuscript is well written and interesting, and it discusses a topic that may be relevant beyond Wellcome Trust programmes in improving doctoral education. Yet in reading the manuscript I noticed elements that raised important concerns which would need to be addressed by the authors. I believe that the manuscript should be revised substantially to address or clarify such concerns. I will go through these concerns in the order that they appear in the manuscript.

 First, in the introduction, the authors describe the findings from Mentimeter word clouds they captured during a taskforce meeting. As a first, yet minor, thought on this point, it would be useful if the colour scheme used in the word clouds could reflect the positivity/negativity/neutrality of the terms, although I understand that this may not be possible to do with fixed Mentimeter results. But my main concern is in the way the word clouds were interpreted by the authors, and I believe that the way they are described needs to be revised to adequately reflect the data embedded in the word clouds rather than a superficial interpretation of ‘bigger words’. In fact, in describing the word clouds, the authors focus on the few ‘bigger words’, disregarding the smaller words inputted in the word clouds. Having used Mentimeter in the past, I know that ‘bigger’ words are sometimes bigger only from 2 or 3 responses using the same term. To be able to judge the positivity/negativity of the students’ input, the full data (i.e., all words and frequencies) should be used. For example, at first glance, I counted approximately 31 negative terms and 26 positive terms in Figure 1. Adapting for the few bigger words and assuming these embedded 2-3 answers, it still means that the authors’ interpretation of Figure 1 as an obvious positive impression may not be justified. I therefore suggest that, if the authors wish to use the word cloud to introduce their work, they should use the meta-data received with these word clouds to interpret their meaning.

 In the methods section, it would be very useful if a copy of the survey was included. I could not find the survey in the online material and making the survey available would without doubt help clarify (although not solve entirely) a few of the points I raise below. I also noted the several use of the adjective ‘novel’ to describe the questionnaire, and wondered whether a less value-laden term could be substituted for the overly-used and badly-defined term ‘novel’.

 Later in the manuscript, the authors explain that “Potential gaps in the training provision for PhD students were considered the most significant investigative aim of the survey…” (p.6 in the PDF version), yet these gaps do not seem to be clearly addressed nor covered by the survey questions. The authors add that the word clouds and text answers helped to identify these gaps, but it was not clear to me where responses really helped identify gaps vs. where they identified impressions and feedback. I believe that this information is in the manuscript, but I would encourage authors to make it more clear where these gaps were identified in the responses of the survey throughout the manuscript.

 Still on p.6 of the PDF manuscript, when introducing the respondents, the authors mention that “Responses were obtained from 36 Wellcome Trust funded PhD students in different years of study at institutions across the UK”, yet it is not until the ‘Other considerations’ section on p. 14 that the reader is aware of the total cohort and, by consequence, the response rate of the survey. I believe that this response rate should be mentioned already on page 6.

 On page 9, figure 6 is identical to figure 8 on page 11. Both figure contain an irrelevant title at the bottom “Most to least preferred mode of training” since both refer to topics rather than to teaching modes. This should be rectified and the missing figure should be added.

 In interpreting Figure 6, the authors also introduce conflicting information about equality and diversity training, stating that “Of 33 respondents, exactly half had not experienced this type of training. However, the students who had received equality and diversity training generally found it useful.” Later in the same section, the authors then state that “The least useful training was commonly reported to be wellbeing training, and equality and diversity training.” Only to mention on page 10 that “Overall, the data shows that students were lacking in training on equality, diversity, and inclusion, although those who had received this training found it useful.” These contradicting findings are very confusing and it would be important for the authors to clarify this, especially in the section ‘Content of Training’. Specifically, it would help if the authors could clarify where the information about the question in which “students who had received equality and diversity training generally found it useful” is captured. Is this from the survey? Is this in an open text question or is it from somewhere else? It seems important that the authors clarify this information and make sure it is reflected throughout the manuscript (including abstract, discussion, and recommendation). If contradictory information was obtained this contradiction should also be explored further and more explicitly. Considering the points above, the summary paragraph before the section ‘Administrator survey’ and sections of the abstract and discussion may also need to be adapted to correspond to the clarifications.

 On page 11 of the PDF version, the authors announce Figure 9 in a section that does not correspond to the elements displayed in figure 9. I believe that figure 9 should be referenced in the penultimate paragraph before the Discussion section.

 In the Discussion section, several conclusions should be revised to better reflect the findings more accurately. For instance, the authors state that “it was nearly universally agreed that training was a good use of time and resources” where this appears to have been agreed by 75.8% of respondents – if I am not mistaken – a high proportion but not one I would qualify as ‘nearly universally’. A bit later, the authors reinstate that “wellbeing training was not prioritised when students were asked to rank the helpfulness of various types of training: it was consistently rated as the least useful” whereas according to Figure 6 EDI training was also rated as the least useful by as many respondents as wellbeing training (see point on EDI above). A few other instances of the discussion section appear to paint a positive outlook that is not entirely reflective of, or not easy to tie to, the results presented. I would thus recommend that the discussion should be revised to clarify the elements mentioned and to transparently reflect the possible drawbacks and possible conflicts in the responses. On a more general note, I would also have liked to see further comparisons with past literature on the training needs of PhD students, and more discussion of the meaning of this work in a broader sense. The manuscript only refers to two references in the discussion section, both seemingly about the impact of COVID in teaching, but I am sure that several studies could provide useful information to complement and enrich the findings from this work.

 Finally, the section about ‘other considerations’ is very helpful, but I had the impression that the authors focused this section mostly on the reasons for the small response rate rather than on what the small response rate may mean for the data. To ensure that the readers understand the strength and limitations of the work, it would be important to expand further on what the data may provide and on where used of these findings should be mindful. For instance, it seems valuable to bring back the issue of the survey not being able to identify the institutions from which the respondents came and to explain whether this impacts the conclusions given the diversity in training and training delivery between institutions. In addition, the authors mention the lack of validation for the questionnaire, suggesting that the study may be used as a pilot for the questionnaire; this point is not reflected anywhere else in the manuscript but may benefit by being added in other sections, especially in the methods section.

 I hope that my comments will be useful to the authors in revising their manuscript. I would welcome them reaching out via the comment section if any of my concern is unclear.

Is the work clearly and accurately presented and does it cite the current literature?

Partly

If applicable, is the statistical analysis and its interpretation appropriate?

Not applicable

Are all the source data underlying the results available to ensure full reproducibility?

Yes

Is the study design appropriate and is the work technically sound?

Partly

Are the conclusions drawn adequately supported by the results?

Partly

Are sufficient details of methods and analysis provided to allow replication by others?

Partly

Reviewer Expertise:

Research assessment; research culture; research careers; research integrity

I confirm that I have read this submission and believe that I have an appropriate level of expertise to confirm that it is of an acceptable scientific standard, however I have significant reservations, as outlined above.

# Response

**Published:** 2023-12-05

**Authors:** Kathryn Sandilands

**Affiliations:** Law School, University of Leicester, Leicester, England, UK

Thank you to the reviewer for your time and comments. We have found this very helpful and have made amendments to the article to address the minor improvements suggested in the peer review. The amendments are addressed in turn below: Unfortunately, we are unable to alter the colour scheme of the Mentimeter word clouds to clarify the designation of ‘positive’, ‘negative’, or ‘neutral’ submissions. As regards the interpretation of the relative size of the words represented in the word cloud, we have included some additional explanation indicating that an enlarged word within the cloud indicates repeated incidence in submissions and have contextualised this within the overall findings regarding the nature of the phrases submitted by students. We have also quantified the ‘positive’, ‘negative’, or ‘neutral’ responses for each word cloud within the report, as well as adding these quantifications to the Figshare collection. An additional citation has been included to enable ease of locating the student and administrator surveys in the Extended data files (Fawcett *et al., *2023b). Where appropriate, the word ‘novel’ has been omitted or altered to better reflect that the survey had not previously been piloted. This is further discussed within the article on page 14 under ‘Other Considerations’. We have clarified that the use of the Mentimeter word clouds were intended to provide overall impressions of student perspectives, while multiple-choice questions permitted more targeted enquiries and free-text responses were encouraged to allow students to elaborate on these issues. The ordering of questions and their different purposes have been added to the methods section to explain how gaps in training were identified from student responses. The response rate to the survey has been more prominently highlighted in the methods section of the article so as to provide context to the reader when approaching the results which follow. The total number of students contacted is now noted at this earlier stage. This is discussed in more detail in the section entitled ‘Other Considerations’ at the end of the article. Corrections have been made to Figure 6 and Figure 8. The axis of Figure 6 has been edited with a more appropriate title. Figure 8 has been updated to include the correct data and legend as described in the text. Both have been updated within the Extended Data files on Figshare, with the collection amended to reflect these changes also. The reviewer noted that there appeared to be a contradiction in the results regarding EDI training. This has been clarified within the article: two questions within the survey asked respondents to reflect on the usefulness of EDI training. In the first, respondents were asked about EDI training independently and half of those respondents submitted a ‘non applicable’ response, indicating that they had not undergone EDI training, while the remainder answered either ‘extremely useful’, ‘somewhat useful’, or ‘neutral’. In the second question, respondents were asked to compare different forms of training and rank them. No option of ‘non applicable’ was offered, which meant that EDI training was ranked as one of the least useful forms of training, though this may not represent the experiences of students who had actually undertaken EDI training. A citation for Figure 9 has been moved within the article to a more appropriate context where its results are specifically discussed. The phrase ‘nearly universally’ has been altered to better reflect the results. More references to the literature have been included, including Williams in the introduction, Meulenders in the results, and Wilson in the discussion. This should enhance the contextualisation of the findings within the article. A review of the discussion section better reflects the drawbacks and potential conflicts of responses. Comments regarding potential future research have been added to the section entitled Other Considerations to better reflect areas which might be improved through further investigation. As well as this, we have sought to contextualise the response rate more clearly in this section. We have also included an additional paragraph to highlight the potential differences between institutions and how this links administrator and student survey outputs, which we are unable to determine. A sentence was also added to the methods section to suggest that this research could act to pilot this questionnaire for future use.
